# Feeling called to care: a qualitative interview study on normativity in family caregivers’ experiences in Dutch home settings in a palliative care context

**DOI:** 10.1186/s12904-021-00868-2

**Published:** 2021-11-27

**Authors:** Maaike M. Haan, Gert Olthuis, Jelle L. P. van Gurp

**Affiliations:** Radboud University Medical Center, Radboud Institute for Health Sciences, IQ healthcare, P.O. Box 9101, 160, 6500 HB Nijmegen, The Netherlands

**Keywords:** Caregivers, Family care, Informal care, Palliative care, Ethics, Caregiver burden, Qualitative research, The Netherlands

## Abstract

**Background:**

Family caregivers, such as partners or other family members, are highly important to people who desire to stay at home in the last phase of their life-limiting disease. Despite the much-investigated challenges of family caregiving for a patient from one’s direct social network, lots of caregivers persevere. To better understand why, we aimed to specify how normative elements – i.e. what is considered good or valuable – shape family caregivers’ experiences in Dutch home settings.

**Methods:**

From September 2017 to February 2019, a total of 15 family caregivers, 13 bereaved family caregivers, and 9 patients participated in one-time in-depth interviews. The data were qualitatively analyzed following a grounded theory approach.

**Results:**

Central to this study is the persistent feeling of being called to care. By whom, why, and to what? Family caregivers feel called by the patient, professionals entering normal life, family and friends, or by oneself; because of normative elements of love, duty, or family dynamics; to be constantly available, attentive to the patient while ignoring their own needs, and assertive in managing the caring situation. The prospect of death within the palliative care context intensifies these mechanisms with a sense of urgency.

**Conclusions:**

Our analysis showed a difference between feeling called upon in the caring situation on the one hand, and how caregivers tend to respond to these calls on the other. Taking into account the inherent normative and complex nature of family caregiving, the pressing feeling of being called cannot – and perhaps should not – simply be resolved. Caring might be something families just find themselves in due to being related. Rather than in feeling called upon per se, the burden of care might lie in the seeming limitlessness to which people feel called, reinforced by (implicit) social expectations. Support, we argue, should enable caregivers to reflect on what norms and values guide their responses while acknowledging that caring, despite being burdensome, can be a highly important and rewarding part of the relationship between partners or family members.

## Background

In the last phase of a life-limiting disease, patients are often cared for by someone close from their social network, such as their partner, grown-up child, or friend.^1^ These people providing family care (Table 1) are pivotal. Due to their often unique relationship with and valuable knowledge about the patient, family caregivers are essential in providing emotional support, communicating with professionals and services, relieving pain and other symptoms, or doing practical tasks [9]. However, especially if the patient desires to stay and die at home, as most people prefer initially [10], the roles and responsibilities of their families and friends are intensified [7, 11] and caregiving may be burdening. This study explores why family caregivers (hereafter: caregivers) persevere, despite the challenges, and which role normativity plays.

**Table 1:** Definition and background of family care in the Netherlands

In this study, family caregiving is regarded as the wide range of aid or assistance in activities of daily living given by an unpaid and untrained person from someone’s direct social network, e.g. a partner, relative, grown-up child, friend, neighbor, or other acquaintance [1–5]. Family care may vary in intensity and duration, but, in any case, goes beyond what can reasonably be expected within the relationship [4]. In the Netherlands, several definitions and criteria for ‘family care’ exist, leading to different estimations of the number of caregivers [6]. Defined broadly, about 35% of people of 16 years and older reported providing unpaid help to someone close with health-related problems [6]. Although the terms are sometimes used interchangeably, we deliberately do not use the term ‘informal care’ to explicitly exclude volunteers from this study. Family care occurs in several settings, i.e. for people suffering from serious illness, long-term mental health problems, or a disability. This study specifically focuses on family care in a palliative care context, as a consequence of metastasized cancer or long-term organ failure. Estimations concerning the number of caregivers in this context are difficult, especially if hand-on help from members of the wider social network, other than the ‘primary’ caring relative, is taken into account [7], which occurs in the majority of Dutch caregivers of terminally ill patients [8]. The lack of a clear-cut point of entering the end-of-life phase further problematizes the estimation [7, 9], as does the observation that not every ‘family caregiver’ recognizes himself or herself as such [3].	

Previous research has highlighted the physical and psychosocial challenges of family care in the palliative phase, recognizing the need for caregiver support [12–14]. Many caregivers live in permanent uncertainty about the future and feel overwhelmed and unprepared for their caring role [15, 16]. Maintaining normality in social engagements can be a struggle, for instance when a caregiver’s sense of togetherness with the patient conflicts with also feeling socially isolated [7, 17]. Caregiving can limit or even ‘chain’ caregivers in their own life and affect their relationship with the patient [16]. About one in five caregivers of terminally ill patients experience a heavy care-related burden [8]. This much-investigated concept of ‘burden’ is reported to be associated with factors like distress at witnessing suffering and disease progression, uncertainty about the situation, role strain, sleep deprivation, spiritual distress, and financial crises [1, 7].

As lots of people feel burdened by caring for someone close, why do they persevere? Many empirical studies focus on predefined outcomes, but little tell us about the individual processes and context that shape the actual care [18]. Care ethical analyses, rooted in feminist studies, have argued that caring is not (only) a matter of one’s free and personal choice. Rather, a caregiver’s agency is deeply tied to the surrounding social and political practices: *“caregivers appear as people finding themselves in a position in which others, they themselves, and also the socio-political context* expect them *to have and take responsibility, as a result of socio-political, personal, affective, contextual, and ethical factors.”* (p. 277) [19] In this article, we are specifically interested in these ethical factors, which we label as ‘normative’, e.g. the normative elements that appear to be essential to the experiences of family members providing care. Normative elements have to do with someone’s convictions about what is ‘good’ or ‘right’ to do, which are particularly relevant when it comes to life and death in the last phase of a life-limiting disease. As Randall and Downie (p.13) argue:*“… any discussion about palliative care occurs against the background of those major questions which relate to the meaning of life and death, or what constitutes a good life (and perhaps death) for a person.”* [20] Normativity has already been recognized as important in palliative care and in family caregiving, but its precise role remains unclear in the caregiving literature. For example, the theoretical Informal Care model proposed by Broese van Groenou and De Boer (2016) understands family caregiving, in general, as being dependent on both contextual factors that influence care provision as well as individual factors that shape one’s intention to provide care, to which they refer broadly in normative terms, i.e. beliefs, motives, values, or norms. Prior empirical studies in palliative care also refer to normative caregiver experiences, such as feeling unprepared yet *responsible* to provide care [15], feeling a general *duty* to care [21], or feeling *obliged* to prefer providing care at home [22].

Drawing on these broad references to normativity, we aimed to specify how normativity shapes the phenomenon of family caregiving within the context of palliative home care. This article’s research question is: how do family caregivers of seriously ill people in Dutch home settings experience caring for their partner or family member in the palliative phase, and how are these experiences shaped by normativity? More insight will provide us with relevant clues about how to better understand and support caregivers.

## Methods

### Study design

Our overall research project aimed at exploring the diverse palette of family caregiver experiences, following the characteristics that are key in the diverging views and philosophical assumptions of a grounded theory methodology [23, 24], i.e. we used an inductive approach, we simultaneously collected and analyzed data while developing theoretical abstractions grounded in the data, we used constant comparison and kept memos, and strived for theoretical sampling. Thus, we ensured a thorough exploration of people’s experiences with caregiving and remained open to both the positive, rewarding experiences that come from caring as well as the experiences of feeling burdened [8]. In line with Straussian grounded theory [23], pre-given concepts or previous studies did not dominate the data collection and analysis to keep an open mind [24–26]. In line with Charmaz’ constructivist approach, however, we considered ourselves as not having an empty head [25]. We started the project with some sensitizing concepts (that were adapted into topics for our interview guides, see Table 2), based on an explorative search of scientific and gray literature, as well as interview expertise in palliative care within the project group (JvG, GO).

**Table 2:** Topics in the interview guides

Caregiver interviews: • Situation and disease process of the patient, (changed) relationship with the patient • A (non) typical day and caregiving ◦ In case of bereaved caregiver: last weeks and time after the patient’s death • Meaning of relationships with others and support • Taking care of oneself, needs, support • Saying goodbye, talking about death, future Patient interviews: • Situation and disease process, meaning of being ill, (changed) relationship with the caregiver • Being cared for, a (non) typical day • Meaning of relationships with others • Saying goodbye, talking about death, future	

During the cyclical data collection and analysis, our background as medical ethicists made us notice normative aspects in the interviewees’ phrasing and overall stories. We became increasingly interested in what motivates caregivers to care for patients in need of palliative care. Therefore, for this article, we specified the initially broad research question of the overall project into how the collected care experiences are shaped by normativity.

### Setting, participants, and materials

Based on the inclusion criteria (Table 3), three groups of Dutch interviewees were included: 1) family caregivers, 2) bereaved family caregivers (within 5 months after the patient’s death) who could describe the last days before and first few weeks after the death of the patient, and 3) patients i.e. people with a life-limiting disease in the palliative phase who received family care. All interviewees were aware that the trajectory of the involved patient was regarded as palliative. We were focused on caregivers’ perspectives primarily. Patient interviews were considered to be complementary data, functioning as triangulation, to better interpret and understand the context of the caregivers’ data. Purposeful sampling was used to include a variety of people, based on gender, age, or caring relationship [24]. Various healthcare professionals (e.g. general practitioners, district nurses, hospital-based professionals) invited potential interviewees, leading to convenience sampling as well. Later on in the study, we strived for theoretical sampling to identify variations and relations in the ongoing analysis [24]. When a family caregiver or patient was interested, MH contacted him or her via telephone, and sent an information letter. The interviews were scheduled within days or weeks after MH first contacted the interviewees.

**Table 3:** Inclusion criteria

All interviewees: • are 16 years old or older; • are mentally competent to engage in the interview; • are fluent enough in the Dutch language to participate in the in-depth interview; • are involved in family care, as either caregiver or care receiver; • are aware that the involved patient is in the palliative phase of life and has a limited life expectancy. The involved patients (not necessarily interviewees): • are 16 years old or older; • have an incurable and life-limiting oncological or neurological disease, organ failure, or elderly frailty (dementia is excluded, because of the specifically changing nature of the relationship between caregiver and patient); • are in such condition that their involved healthcare professional would not be surprised if this patient died within the next 12 months (e.g. the *“surprise question”* as used in palliative care); • receive family care at home from at least one loved one (partner, child, parent, sibling, friend, etc.); or has received this care before being transferred to a hospice or other care institution.	

The interview topics (Table 2) and questions were reviewed by various experts in qualitative research and palliative care. We constructed different guides for the different interview groups, that were adapted several times during the data collection to deepen the ongoing analysis and achieve saturation. First author MH received interview training from an independent senior researcher with ample experience in qualitative research and personal experience with family caregiving. The interviewing was piloted with the trainer twice.

### Data collection

MH conducted the interviews. Although the interviews were guided by general topics (Table 2), they were minimally directive. Probing was mainly based on the situation and what the interviewee said. Field notes and memos were made to facilitate the iterative process of interviewing and analyzing. The interviews were audio-recorded and transcribed verbatim. The original Dutch quotes for this article were translated into English.

### Ethical considerations

Ethical approval was sought from the Medical Review Ethics Committee region Arnhem-Nijmegen (registration number 2017–3415), who determined that this study does not fall under the scope of the Medical Research Involving Human Subjects Act (WMO). Potential interviewees verbally consented to be contacted by MH. All participants gave their written informed consent, after receiving an information letter. Because healthcare professionals acted as our gatekeepers in finding participants, the potential interviewees’ privacy was an important point of consideration. In addition, given the interviewees’ vulnerable position, caution was needed in inviting and interviewing people. The possible emotional or physical burden of the interview was acknowledged both in the information letter and the interview itself; people could withdraw at any time. Furthermore, because the interviews involved personal and emotional topics and we did not want to disturb existing relationships, MH tried to conduct the interview individually without others nearby – unless the interviewee wished differently or practical circumstances did not allow this. Every participant received a numerical code and all interview transcripts were anonymized. The data were safely stored, as was stated in an approved data management plan.

### Data analysis

Following an iterative approach, characterizing grounded theory, the data were analyzed throughout the collection process [27, 28], using ATLAS.ti software. MH first coded transcriptions individually and constantly compared the analysis to previous codes, staying close to the language used by the interviewees [25]. As the cyclical process of grounded theory allows, rereading, coding, and collecting new data occurred simultaneously to dig deeper into the research question [25]. In line with Straussian grounded theory, open, axial, and selective coding were used to find categories, overarching themes, and, ultimately, patterns [23, 24]. Meanwhile, hunches and decisions concerning codes, categories, and patterns were kept in memos [26, 29] to add to the transparency and credibility of the research. Several visual displays were made to organize ideas and discuss the theory [29]. The data collection continued until new interviews no longer provided new insights on general patterns within the group of caregiver participants. Although the use of theoretical sampling was limited, we then decided that saturation was reached.

### Analytic rigor

In contrast with a classical view on grounded theory [23, 29], we followed Charmaz’ constructivist approach in regarding the researcher as not being neutral or distinct from the research process [25] and stressing the importance of a researcher’s reflexivity conducting grounded theory [23]. Thus, for validity reasons, JvG independently coded the first three interviews as well [25, 27]. We critically discussed our differences as coders without looking for a compromise. During the interviewing, MH verified her interpretations with the interviewee by summarizing and choosing multiple angles during the interviews. General patterns and themes were checked to employ specific interview questions in later stages of the data collection. Moreover, in June 2018, the identified themes and patterns in the data analysis were discussed during a feedback meeting with five interviewees. In addition, the authors regularly discussed their results with a sounding board of experts, mainly from various stakeholder organizations. Utilizing memo writing, but also discussion and checks both within and outside the project team, measures were taken to ensure MH’s reflexivity and critically consider her role and possible biases (for example, focusing on challenging aspects of caregiving in the interviews). Throughout the study, ongoing peer review and discussion between all authors were essential.

## Results

From September 2017 to February 2019, a total of 15 family caregivers, 13 bereaved family caregivers, and 9 patients participated in one-time in-depth interviews (Table 4). All caregiver interviewees were (one of) the involved patient’s primary family caregivers. Six interviews were conducted with two interviewees present (caregiver and patient, or two caregivers), due to their preference or initiative. In all interviews, the main subject of conversation was family caregiving for a patient in the prospect of an approaching death. Life expectancies of the involved patients varied from weeks, months, to maximally a few years. Most interviewees were interviewed individually at home or where they resided; two caregivers preferred to be interviewed without the patient nearby and were interviewed at the researchers’ department. Interview duration ranged from approximately 38 min to 2 h and 27 min.

**Table 4:** Participant characteristics

*Characteristics*	Family caregivers (*N* = 28)	Patients (*N* = 9)
Male / Female	12 M / 16F	4 M / 5F
Age	23 to 84 years (mean 58.1)	61 to 95 years (mean 74.2)
Retrospective interview	13	not applicable
*Relationship between patient and primary family caregiver*		
• Partner	18 partners	7 partners^a^
• Child–parent	9 children (1 son)	1 parent^a^
• Other family member or friend	1	2
*Diagnosis*		
• Cancer	16	5
• Organ failure (e.g. chronic obstructive pulmonary disease or heart failure)	6	2
• Other, comorbidity or unclear condition	6	2

^a^One of the 7 partner interviewees also received care from her child and specifically told about that in the interview

### Overview

**Fig. 1:**
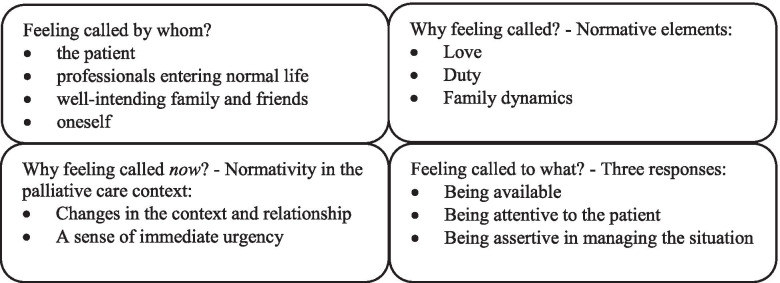
Overview of the presented analysis about family caregivers feeling called to care

Our results present a qualitative analysis of the phenomenon of family caregiving in palliative care, as seen from the perspectives of various caregivers and patients, focusing on how normativity shapes caregiving behavior (Fig. 1). Central to this study is the persistent and sometimes continuous feeling of *being called to care*. Our analysis further explains a pattern in this feeling: by whom, why, and to what?

Caregivers feel called either by the patient’s explicit or implicit calls for help, professionals entering normal life, well-intending family and friends, or by oneself. Their responses to feeling called seem to be evoked by general normative elements, e.g. love; duty; and family dynamics. More specifically, by subjecting relationships to pressure and intensifying the felt calls with a sense of urgency, the palliative care context seems pivotal in understanding to what caregivers feel called, e.g. being constantly available, attentive to the patient while ignoring their own needs, and assertive on several fronts in managing the caring situation. Our analysis, thus, revealed a difference between feeling called upon in the caring situation on the one hand, and how caregivers tend to respond to these calls on the other – reinforced by normativity.

#### Feeling called by whom?

We found a persistent and always-existing urge which we identified as the manifestation of *feeling called to care*, this study’s central theme. Looking at the caregivers’ stories from an ethical perspective, this feeling presents itself as pre-reflexive in their stories, e.g. not based on a well-considered choice using explicit moral arguments: caregivers often felt a strong urge to act and automatically did so, yet without having been able to thoroughly reflect on why or how*.* Some people reported feeling what was needed intuitively because they knew the patient so well and the two of them spent so much time together. Feeling called to care manifested itself in the caregivers’ experiences in four ways: by the patient; professionals entering normal life; well-intending family and friends; or one-self.

##### Feeling called by the patient

The family caregiver was often the person closest to the patient. Caregivers felt called by patients explicitly, when they needed something to eat or drink, asked for assistance with going to the toilet, expressed anxiety and worries, felt ill, or even screamed in pain. Patients were fearful and stressed when they were left alone, some patient interviewees tried to maintain in control by having (multiple) phones within reach, frequently asking or calling their caregivers. As a result, caregivers experienced a, sometimes continuous call:

*... I have the feeling that I am being deeply called upon to do so, that it is 24-hour care. Really. [...] It starts in the morning when I get up. The first thing I do is put her on the bedpan. [...] It goes on until she goes to bed; it is continuous. Sometimes you can have 15 minutes or so, a few minutes, or if someone is there, you don’t even have that. But it is actually always there. [...] Every moment is an appeal, every moment. ... (i17, husband caring for his wife with a lung disease)*

This was endorsed in retrospective interviews: some bereaved caregivers felt relieved by not having to continuously respond to the urgent needs and suffering anymore, which showed the urge of feeling called explicitly.

Next to explicit calls, patients showed implicit calls, for example in their preference for and dependence on the family caregiver being the primary person to provide care:

*Patient: Family﻿ care is everything here right now. Without it, there is nothing left. [...] Interviewer: Why do you think it’s so important that she [his wife] gets more help? Patient: So she can carry on, eh? For herself. For her daughter. It sounds selfish, but also for me, eh? Because if she fails ... Tell me ... (i8, male patient with cancer being cared for by his wife)*

##### Feeling called by professionals entering normal life

In caring at home, caregivers also had to deal with professionals, technology, or aids. Usually, they were untrained medical laypeople, feeling overwhelmed by disease symptoms:

*… he was in pain and felt he couldn’t get air. I have no medical background or anything, so. [...] It is like being in an unfamiliar forest, so you don’t even know what is there. (i11, wife caring for her husband with cancer)*

Professional home care, then, could offer relief by taking over caring tasks and responsibilities. At the same time, professionals’ presence did not seem to resolve the aforementioned patient’s calls. People would still feel alert and called to provide care themselves:

*Daughter: And the best thing about it [interviewer name], that my father called me after all. “[Daughter’s name], where are you?” So I had to get up. Because I heard my father, and then the girls [from the night care] said, “no, just stay put”. I say “yes, my father calls me, never mind. I’m awake anyway”, I say.*

*Son-in-law: Yes, and he does not understand Dutch well either.*

*Daughter: No, and then he started talking [foreign language] to those girls too. (i21, bereaved daughter and her partner caring for her father with cancer)*

In addition, the presence of professionals instigated a call itself in several ways. For some people, it was difficult having strangers in their home several times a day, taking-over normal life: the *“circus”* of professional caregivers stopping by according to one interviewee. Caregivers entered what an interviewed son said to be the *“new world which is called care”*, sometimes accompanied by bureaucracy. Negative experiences with institutional care or the tight schedule of professional home caregivers also urged family members to provide the care themselves. Being able to organize care in the way one chooses (e.g. time or place) was seen as an advantage of providing family care instead of waiting for professional help.

##### Feeling called by well-intending family and friends

Caregivers and patients were often surrounded by family or friends, by whose attention they felt supported and loved. Despite being well-intended, their visits, how-are-you-questions, worries, or advice could instigate a call for caregivers, notably when mediated by social communication technologies demanding a continuous presence. Some patients found it difficult to maintain their autonomy. Having to tell the sometimes confronting truth over and over again could be difficult for caregivers. Yet, according to some, by explaining the situation regularly, it also lost some of its emotional edges. In a way, the disease seemed to take over normal life and conversations:

*But often you also have to talk about the disease before you can talk about other things. So when I join in – I play tennis once a week with a friend, a tennis mate. First I talk to him for 10 min about [wife’s name] and the disease. Then we can play tennis. [...] And if you have had that, say, once you have bitten through the sour apple, then you can also enjoy the rest. (i14, husband caring for his wife with cancer)*

In some family contexts, talking together about death was taboo, resulting in a tension between respecting one’s family norms vs. one’s own desire to discuss last wishes, the upcoming funeral, or other end-of-life issues.

##### Feeling called by oneself

Lastly, feeling called to care could be self-imposed. Caregivers sometimes felt like they were the only, or the most capable, person in the family to arrange things, wanted to do things themselves, or specifically asked what the patient wished:

*Yeah, maybe I’m in that [appeal] too, because I’m going to ask him, “what would you really like to eat?” [...] That appeal, I pull it towards me. It is not that he says “you have to do that for me”. I ask him what he would like. (i29, son caring for his father with aneurysm)*

#### Why feeling called? – Normative elements

What motivates people to respond to these calls? This section explores normativity essential to the caring situation and relationship with the patient. We found several, often co-existing, normative elements that evoked caregiver responses: love, duty, and family dynamics.

##### Love

First, caregivers felt called to care for the patient out of love or a special bond:

*Yes, you just do that for each other. [emotionally] He is my husband you know, so I love him. You don’t want someone else to do all that for you. (i28, wife caring for her husband with cancer)*

Caring out of love could be based on a promise, for example between partners (for better or worse) or other family members. Caring was also seen as self-evident in a loving relationship or was valued because it enabled people to live their normal life as much as possible. One patient noticed that being cared for by her partner was different from being cared for by her son because the latter was less evident (*“he is still my child”*). According to several daughters, love is what makes family care different from professional care. Caring could also be expected because the patient only trusted family members, or it stemmed from reciprocity in a loving relationship: for some, it was important to imagine themselves in the situation of the patient to make decisions. However, siblings sometimes had conflicting opinions about what was best or most pleasant for the involved parent.

##### Duty

Caring out of love sometimes co-existed with feeling a duty, for example keeping a promise regardless of whether love was still felt. Care could also be provided out of obligation or duty in the relationship or in the wider family:

…*, No, I don’t have t*o *be put in the limelight because this is just a part of my job. It’s a piece of moral duty that belongs to the fact that I am a daughter of my mother who took care of me. (i13, bereaved daughter caring for her mother with comorbidities)*

This obligation stemmed from social expectations concerning familial relationships or helping others in need. Important to note here is that we only interviewed caregivers in the Netherlands and did not specifically ask for demographic information about culture or religion. Amongst interviewees who described their culture as family-oriented, we found a moral duty to take care of one’s parents in return for their upbringing. An interviewee, however, emphasized love and honor as motivating in caring for her father, instead of this norm of reciprocity or gratitude in her Islamic culture. The value of helping others in need, which was sometimes religiously inspired, was also found amongst interviewees with an intuitive intention or moral obligation to help whenever they could.

##### Family dynamics

Especially in parent-child relationships, the sometimes troubled family history and the specific relationship with the parent played a role in what caregivers expected of themselves and how they responded in the caring situation. For example, being the most responsible sibling, being the darling sibling of a mother, or the past conflicts between family members. In addition, contrast experiences with another family member’s death or with caregiving in the past were directive in persevering:

*… “I will take care of him until I drop, and then I can’t do it anymore”, I say. This will never happen to me the way it did to my mother. [...] Fortunately, I have managed to accomplish it. (i19, bereaved wife caring for her husband with a neurological disease)*

A family’s hierarchy also influenced caregivers’ feeling of being called to care. Having a specific position within a family, such as being the eldest or being a professional healthcare provider, could lead to being assigned to the role of primary caregiver by one’s siblings.

#### Why feeling called *now*?* –* Normativity in the palliative care context

To some extent, the aforementioned calls and normative aspects apply to all types of long-term family caregiving at home. What, however, is markedly different in a palliative care context is the décor of deterioration and the prospect of an approaching death. This section explores normativity within this specific context.

##### Changes in the context and relationship

Our results showed the context to be changing and increasingly palliative, consisting of a poor prognosis and deterioration, i.e. physical changes and/or a loss of autonomy while handing over things and becoming increasingly dependent on care. Some patients feared dying alone, choking or in serious pain, or worried about what they had to leave behind. In partner relationships, the inevitability of death could lead to feelings of loneliness, being unable to live and share (sexual) life as they used to, or having to find proximity in other ways, and no longer sharing a joint future. The deterioration and fear seriously-ill people experienced increasingly changed them into a ‘patient’ in the eyes of their partner or family member: the person slowly *“faded”*, for example, and was no longer always recognizable as he or she used to be. These changes invoked a role shift for the partner or family member as well, who became more or less, sometimes in an unwanted way, a ‘caregiver’ with new responsibilities:

*Yes, no, you don’t want that. You just want your partner to be your partner. Often that’s what she is too. If I have things to deal with, then she is still the person I go to, the one who gives me advice. I always want to hear what she thinks about it. We can level with each other, we can spar with each other. But there are just considerable times when I am home help for her. (i14, husband caring for his wife with cancer)*

##### A sense of immediate urgency

The prospect of nearing death, which changes the relationship, also leads to a sense of urgency in responding to felt calls: caregivers believed they had one final chance to do the right thing for the patient. This seems pivotal in understanding what motivates them to immediately respond to calls, sometimes regardless of whether this is rewarding or physically or emotionally burdening:

*…that’s what I said, I only have one chance of doing this for him. And that is simply why I am putting my back into it and why I am doing it. (i29, son caring for his father)*

#### Feeling called to what? – Three responses

Our results showed that the aforementioned normativity within the context urges caregivers to be constantly available; attentive to the patient first while ignoring their own needs; and assertive on several fronts of their lives in managing the caring situation. Despite the dilemmas described below, it is important to note that intensively caring for the patient also evoked *positive* feelings among caregivers. Although bereaved caregivers acknowledged exhaustion, they spoke of being “grateful” for having persevered and having enjoyed precious moments together, feeling *“honored”* or *“proud”* to have been able to provide family care. Some spoke of a closer and more intense relationship, for instance by spending more time together and cherishing small things or meaningful moments as the *“last things”* in the light of the approaching death.

##### Being available

In reaction to both the urgency and insecurities in a worrisome palliative care situation, as well as the patient’s fear of being left alone, caregivers strived to be always available, reachable, and alert – live or by phone. Some caregivers felt *“imprisoned”* in their home, feeling obliged to be constantly stand-by for help. Leaving the house was easier when someone else stayed with the patient. Yet, overall, caregivers found it hard to let go of the situation and not think or worry about it, even when the patient was out of sight or when they themselves were out of the caring situation for a moment to get groceries, see friends or family, take part in sports, or work, especially with other people’s questions. This illustrates the pressure of feeling called: despite leaving the situation, caregivers still felt occupied. Some bereaved caregivers experienced feelings of guilt for not having been present all the time:

*Interviewer: … You said that it was painful for you that you were not there with him at the last moment.*

*Sister-in-law: Yes, because I left him alone […] while I knew he was so afraid. Because I assured him, “[name of brother-in-law], when the time comes, I will take care that I am there with you.” But this was unforeseeable. (i22, bereaved sister-in-law caring for a male patient with chronic obstructive pulmonary disease)*

Being medical laypeople, caregivers were sometimes unable to take away pain or breathlessness. Some told of situations in which they stood by helplessly or actually preferred to not see the patient’s suffering, which also shows the tragedy of being present all the time.

##### Being attentive to the patient first

We found a persistent tendency among caregivers that everything should be about the seriously ill patient, leading to self-ignorance, e.g. meeting the needs and wishes of the patients first, before addressing their own. The disease literally took over caregivers’ normal life in their own house: medical aids on the dinner table, a bed in the living room, or shelves full of medication. Work, volunteer jobs, social activities, or trips were put on hold in light of care or spending time together. Caregivers sometimes felt lonely in their daily struggles, or frustrated about the lack of attention from professionals for their perspective:

*… but a family doctor like that who never says, “How are you doing? Can you manage?” […] you think it’s normal, you don’t know any better. You think, it’s not about me; it’s about papa. All the while I was the pivot. (i31, bereaved daughter caring for her father with cancer)*

Caring could result in physical exhaustion, but several people also pointed to what we have identified as a normative pressure stemming from being called in light of an inevitable death:

*Yes, it was tough, really tough. And I don’t mean that I was physically tired, but you reach a point where you are in a sort of tunnel, and you just go on and on. Well, also later on, you think ‘my God, phew’. But there is simply no going back from there, in the sense that you want closure in a proper way. (i25, bereaved husband caring for his wife with cancer)*

The feeling that one can never turn back the clock again was said to be motivating:

*… I can get up in the morning […], I look in the mirror, I feel no guilt because I have done everything, and you can’t turn back the clock. So, in a loved one’s last phase, you have to try to do everything you can, try to do what you would like to do. Because after that, you can’t do anything, and you could regret that. (i21, bereaved daughter caring for her father with cancer)*

Balancing attentiveness to the patient with leading one’s own life could cause dilemmas for both patients and caregivers. Patients sometimes worried about being a burden for their families, acknowledging their needs and preferring to not restrain them in their activities outside the caring situation. Some caregivers would not express their burden to the patient to spare him or her from feeling guilty. Having to raise young children but not being able to share this anymore with a seriously ill partner, could further problematize finding a balance. Overall, patients, as well as caregivers, seemed to want to respect each other’s wishes, even if this conflicted with their own, for example about (not) having open conversations about death, arrangements, or last wishes.

Societal expectations played a role in some dilemmas. On the one hand, going out and engaging in fun activities could lead to a feeling of having to explain one’s behavior to other people, showing the persistent norm that everything should be about the patient. Bereaved caregivers sometimes experienced feelings of doubt, seeking confirmation that they had done it right and had given enough. On the other hand, a caregiver’s large amount of time invested in caring or a self-sacrificing attitude could evoke critical questions by friends or family as well:

*To fight the resistance of others [raises her voice]: “Oh, it’s irresponsible.” “You are sacrificing yourself and you have nothing left.” […] It felt as if I had to justify everything while I know very well what my responsibility is. My responsibility is not somebody else’s responsibility, so there. I know what’s best for him, what’s the most fun, what he likes most. ... I focus it all on that. (i19, bereaved wife caring for her husband with a neurological disease)*

##### Being assertive in managing the situation

While being constantly available and attentive, caregivers felt demanded to uptake an assertive attitude on several fronts of their shared lives, as a response to the diverse calls in the caring situation.

Concerning practical caring duties, caregivers often referred to themselves as *“manager”*, e.g. doing household tasks, preparing and sometimes administering medication, accompanying the patient to doctors’ appointments, informing family and friends and managing visits, maintaining contact with organizations and healthcare professionals, arranging for medical aids and adjustments to the home setting, or doing the record-keeping and managing accounts. They also had to assertively call for help in time. Sometimes, a *“battle”* was fought with organizations or professionals, with caregivers being the patient’s spokesperson.

Assertiveness could be required in the caring relationship itself too, in confrontation with deterioration. Some partners motivated their loved ones when they themselves no longer could, made jokes or had a laugh to make it bearable:

*Because I was relatively stronger than the rest of the nurses. I picked her [his wife] up [to put her on the bed pan] and she went with me. I said “Let’s go dancing”, and then I maneuvered a bit. “A lovely dance.” Then she had to laugh again. We had quite a bit of fun with everything. (i27, bereaved husband caring for his wife with a lung disease)*

Some caregivers found it hard to do nursing tasks such as washing because they had difficulties recognizing the naked and vulnerable patient as the partner or parent he or she used to be. For others, the relationship history guided their behavior, for example in taking care of a father who had always been dominant and harsh. Assertively *“switching the button”* from child role to caregiver role was helpful for some daughters to be able to do these tasks. While managing all kinds of practical caring activities could be difficult and tiring, for some, the burden of care lay in relational aspects:

*I can discuss that with my husband, but sometimes you just notice – oh, what am I talking about? [...] I think, you should see, he looks so sick now, but I start talking about it [son in puberty] who once again did not get up [out of bed], yeah. Those are just normal things that are quite difficult for me. More difficult than cleaning or putting out the garbage cans. (i16, wife caring for her husband with cancer)*

An assertive attitude would also be demanded when several family members jointly provided care for their parent, and conflicts arose: what is best for our mother? Family caregiving could cause fights among siblings, with every child having an individual relationship with the parent while balancing their private life and needs. Sometimes, disappointments arose about the lack of involvement of other family members.

A last possibly tensed front requiring assertiveness was having a (volunteer) job while providing family care. Working and having contact with colleagues could be helpful, but some caregivers were not able to concentrate on tasks as before. Taking time off or receiving caring leave could be difficult. Some caregivers felt that they had to justify the fact they wanted to take sick leave to care for the patient themselves, instead of asking a neighbor:

*I certainly felt abandoned when I had to defend myself before I could be present at the chemo days. I got remarks like “Do you really have to be there?” “Can’t a neighbor or a good friend do that?” [...] It is, of course, completely ridiculous to say to someone who has just had very bad news, “Ask if the neighbor wants to go with her”. (i12, bereaved husband caring for his wife with cancer)*

Overall, some interviewees wondered what it would be like for other caregivers, especially the less assertive ones or people with a migrant background:

*…I am outspoken, I speak the language well, I use the medical terms correctly; and even then I don’t get anywhere. How about the others who are not from the western world. [...] There are standards that you have to meet. If you don’t fit, you are left out. (i31, bereaved daughter caring for her father with cancer)*

## Discussion

This article presented a qualitative analysis of experiences with palliative family caregiving in Dutch home settings, focusing on the role of normativity. In sum, we first showed by whom caregivers feel *called to provide family care*, this study’s central theme*.* Then we showed why people feel called, emphasizing how love, duty, or family dynamics motivate caregivers and in what way the palliative care context intensifies feeling called upon with a sense of urgency. These motives, lastly, explain to what people feel called.

### The limitlessness of feeling called to care

Feeling called to care starts not only with the obvious cause, e.g. a patient’s need for care as the Informal Care Model [18] suggests, but can also be instigated by health care professionals entering a caregiver’s normal life, well-intending family and friends, or it can be self-imposed. We will elaborate on this self-imposed call in the next section. Concerning the call instigated by professionals: previous research shows how professional home care provided security, allowing caregivers and patients to focus more on their family life and prepare for death [30], and offered relief when the right assistance was given on the right time [31]. Our findings confirm this, but also show how professionals entering normal life and routines can instigate a call towards family caregivers, in line with the finding that support sometimes overwhelms or adds responsibilities [31]. Our study also reinforces the idea that previous negative experiences with the healthcare system are motivating the provision of care at home [31], thus showing how caring practices are related to their sociopolitical context. Furthermore, concerning the call instigated by family and friends: caregivers’ often close relationship to the patients and their gatekeeper role places them in a complex social web, highlighting the challenge of maintaining relationships during and beyond the dying process [32], in line with what we found to be a call by well-intending but sometimes demanding friends and family surrounding the caregiver and patient.

Although our results show that caregivers often feel constantly called upon, it should be emphasized that this does not necessarily equal feeling burdened. Caregivers often express ambivalent feelings: caring can be a positive, rewarding, or honoring experience, *while* at the same time feeling occupied or exhausted. In the light of sharing last moments and activities, specifically, bereaved caregivers in our study experienced gratitude for the time spent with their partner or family member, and for having been able to persevere and to give everything they could. Previous research also suggests that although feeling burdened by caregiving in a palliative care context, caregiving can help appreciating ‘the little things’ and becoming closer to the patient [8], and may be a personally meaningful and transforming experience [33].

From each of the found appearances of feeling called, it becomes clear that caring situations are, as care ethicists argue, *“situations that are given to us, that we find ourselves in, as a consequence of being related”* (p. 527) [34]. The families in this study often found themselves embedded in practices in which they felt called and responsible to care [19]. Our results suggest that – rather than in feeling called per se – the burden of care might lie in the seeming *limitlessness* to which people feel called, i.e. the self-ignorance that lies in being constantly alert and available, attentive to the patient first, and assertive in managing the situation while also facing dilemmas in balancing care with one’s other needs. This resonates with the previously investigated all-consuming nature of caring in a situation of serious illness, overwhelming people with its demands [35], *“being on 24/7”* (p. 1232) [31], and with the observation that family care has an impact on a caregiver’s whole personal realm, i.e. feeling the physical and emotional burden of home care and experiencing limitations in living normal daily life [16].

Previous research used existential psychology to understand the psychological complexity of caregivers’ emotional challenges [36]. Our study, however, adds a further interpretation of caregiver responses to the felt calls, as they seem to be reinforced by the *normativity* essential to the actual caring situation. We suggest that it is the inherent normative nature of palliative family caregiving that invites us to rethink our concept of burden and to challenge our bias towards the negative aspects of caregiving [37, 38].

### Normativity and social expectations in a palliative care context

The specific, e.g. palliative, care context seems crucial for understanding caregiver experiences, as it changes relationships [16] and intensifies the felt calls with a sense of urgency of having only one final chance to act. The assertive response to the felt calls we found resonates with findings on taking charge in coordinating home care and making important decisions, in which bereaved caregivers often *“felt thrust into this role without adequate recourses to fulfill its expectations”* (p. 1232) [39]. This reference to expectations underlines how normativity shapes caregiver experiences.

In addition to this specific context, the general normative aspects of love, duty, and family dynamics appeared to be a motivating force behind what caregivers expect of themselves. Interestingly, in family caregiving for people with dementia, the same aspects were found to be interwoven, paralleling the long-term and fluid caregiving role in that context [40]. That long-term care role was found in our study among participants caring for someone with end-stage chronic obstructive pulmonary disease, as opposed to a role that was more suddenly imposed and more directed towards an imminent death among participants caring for someone with metastasized cancer. Previous studies in palliative care also implicitly referred to normativity, for example in caregivers being determined to care for their loved one at home out of *“love, respect, obligation, or giving back to someone who had given them so much and as a way to honor their ill family member’s wishes”* (p. 1232) [31]. This article, however, focused on this normativity explicitly, aiming at providing a more complete overview of the normative aspects motivating caregivers.

A sociological perspective helps deepen our understanding of how these normative aspects are shaping caregiver experiences. Ultimately, family caregiving is an evolving experience, subject to social scripts and expectations [32]. According to sociologist Hochschild’s framework about how people make sense of their emotions, we all live by ‘framing rules’ that govern how we view our situation, and by ‘feeling rules’ with which we relate to these frames and define what we should or should not feel [41, 42]. Caregivers, seen from this perspective, judge their experiences and feelings based on how well they fit with what they believe is to be expected from a ‘good caregiver’ [32]. Misfits inevitably lead to feelings of failure, Broom et al. (2019) argue: caregivers’ actual but perhaps ‘inappropriate’ feelings then conflict with their intentions and, sometimes even romanticized, expectations, such as ‘till death to do us part’. To a certain extent, these sociological mechanisms showed up in our study. Caregivers used such expressions as ‘till death do us part’ or ‘for better or worse’ as a motive to persevere while feeling exhausted, or they wanted to keep doing everything they could to prevent themselves from not feeling guilty afterward (the feeling of failure in Broom’s terms). Or they regretted not having been able to be present when their family member died, showing the *“inevitable messiness of the dying process”* (p. 7) which obstructs caregivers’ abilities to achieve their desired outcomes [32].

However, notably, we did not observe many explicit social scripts or expectations in our participant’s stories. In comparison with the other three manifestations of feeling called, the fourth – the self-imposed call, stemming from one’s convictions and values, seemed less thick. Perhaps, participants could or did not articulate these social expectations by themselves. This can be explained by the fact that our interview questions were focused on *personal* experiences and more on the relationship between caregiver and patient while less on the phenomenon of caregiving in general*.* Our results might imply that the interviewed caregivers were motivated purely intrinsically, as their limitless caring behavior would also suggest; or that these caregivers did not recognize the social expectations as motivating. This would imply that social scripts and pressure play a more implicit and subtle role.

Further research is needed to investigate a possible difference between reacting to a felt call out of intrinsic motives or because of what is expected. Future research specifically focused on normative aspects might benefit from a serial exploration. Interviewing the same caregivers serially, both before the death of the patient and during bereavement, might allow for showing ambivalent feelings that are suppressed in the dying process and can only be revealed in the bereavement phase [32].

### Practical implications: rethinking caregiver support

Studies sometimes show average or median hours of family caregiving per week to indicate burden [11], but invested time is not the only issue nor the most relevant. Caregiving is complex, as we have shown by providing more insight into the inherent normative nature of family caregiving, and is also deeply connected to one’s social, cultural, and political context and related feelings of power [19]. Revealing this complexity to caregivers gives counterweight to dominant expectations [32], and may enable them to talk about their seemingly inappropriate experiences or feelings – whether negative or positive. This article’s insights also provide us with relevant clues about how to better understand and support caregivers.

As our study showed, caregivers want to stay close to the person they care for, reinforced by normative aspects. In our belief, the related pressure will not likely be resolved by professional home care or respite care. Respite might offer effective relief, provided that it is adjusted to caregiver needs, for example concerning confidence about the patient being in good hands [31]. However, reduction of caring hours or organizing activities outside the caregiver’s home to take time-off might not be the only suitable solution for people who do not wish, dare, or feel able to leave their loved one. It was already suggested that being both relative and family caregiver might create a reluctance in asking for professional help, due to the dynamics of these sometimes conflicting dual roles [15]. Our results help us understand this reluctance. Feeling called upon or even burdened does not necessarily mean that caregivers do not want to provide care anymore, or that they wish support or respite. Taking into account the always-existing normative and complex nature of caregiving, support should not be aimed at liberating caregivers *from* the situation but supporting them *in* whatever overwhelms them in providing family care.

This implies a responsibility for healthcare professionals regarding the already widely acknowledged need for better collaboration between family caregivers and supporting professionals in the palliative home setting [9]. They should recognize that caregivers might be determined to enable their partner or family member to die at home, regardless of whether this is burdensome, and align their support with caregivers’ own goals in caring at home [31]. In addition, support should enable caregivers to pause and reflect on (and perhaps break) the social rules implicitly guiding their sometimes automatic responses concerning what is expected or appropriate [41]. Although we acknowledge that caring might be something families find themselves in (*“you just do that for each other*”, as an interviewee stated) and a caregiver is never a freely choosing person [19], we believe there is some degree of agency in how one provides family care. As our analysis showed a difference between feeling called and how people respond, we suggest that caregivers should be enabled to explore the feeling of being called upon – by whom, to what, and why –, to make them aware that, to some extent, they *do* have choices in how to respond. Such exploration helps to address their immediate and sometimes limitless caregiving behavior but also allows for tailoring support to individuals’ needs. After all, family caregiving, regardless of being burdensome, can be an integral, and for some highly important and rewarding part of the relationship between partners or family members.

### Strengths and limitations

As many studies focus on either current or bereaved caregivers [32], a strength of our study is that both were interviewed. In the bereaved group, however, it was difficult to distinguish between grief and the impact of the withdrawal of caregiving. The fact that we also interviewed patients has broadened our analysis. Another strength is that our analysis was conducted cyclically, and co-occurred with collecting the data.

A limitation is that it is likely that we have only interviewed people who felt able to talk about their experiences and thus consented to participate. We cannot rule out the possibility that certain caregivers were excluded from our sample, for example severely overburdened caregivers, or partners with a non-loving relationship affecting one’s caregiving behavior and feelings. Theoretical sampling of such participants, although important for a grounded theory, turned out to be practically difficult, due to the unsteady and precarious circumstances of the study population, time constraints, and general difficulties in finding participants. Theoretical sampling with regard to the interviewees’ personal circumstances (such as being deeply religious or having a troublesome relationship) was hindered by practical difficulties and privacy related issues. It was also difficult to reach interviewees with migrant backgrounds. The ratio of daughter/son in the sample of children who cared for a parent was relatively uneven as well, but at the same time reflects the dynamic of gender in care provision, as recognized in palliative care for older adults [43] or among people with a migrant background [41]. A further limitation in this regard is that we did not systematically document demographic variables of each participant, such as religion or social background, but left it to the initiative of the participants whether or not to share such information.

Furthermore, the interviews likely prompted participants to give words to their experiences, as interview research often provides people with an opportunity to reflect on their situation [44]. This can be regarded as both a strength and a limitation. Normative aspects especially are often implicit until made explicit by ethical reflection or questions; our participants might not have put this normativity into words by themselves. In addition, it can be hard for people to articulate social expectations [32] or express certain ‘background worries’ occupying their minds in light of more pressing matters in the palliative care situation [45]. A strength, in this respect, was our background as medical ethicists, leading to sensitiveness to normative aspects in caregivers’ daily speech, although we did not specifically ask interviewees about this.

## Conclusions

By providing more insight into the inherent normative nature of family caregiving of seriously ill patients within a palliative care context, we have added complexity and depth to our understanding of the much-investigated concept of caregiver burden. Our study indicates that feeling called to care, as far as it is burdensome, cannot – and perhaps should not – be resolved. Caring might be something families just find themselves in due to being related. Rather than in feeling called upon per se, we believe the burden of care lies in the seeming limitlessness to which people feel called. Social expectations play an important, yet often subtle and implicit, role here. Support, then, should enable people to explore the feeling of being called upon – by whom, to do what, and because of which norms or expectations. This helps to tailor support to individuals’ needs. Family caregiving, regardless of being burdensome, can be an integral and for some highly important and rewarding part of the relationship between partners or family members.

## Data Availability

The datasets used and/or analyzed during the current study are available from the corresponding author on reasonable request.

## Footnotes

1 We as authors are aware of the debates about the use of the term ‘patient’. We acknowledge that, especially within the context of this study, people are foremost each other’s partner, parent, friend, or other family member. For the sake of clarity, however, we use the more formal term patient for the person who receives family care.
